# Brugada-like ECG Changes After Conducted Electrical Weapon Exposure: A Case Report

**DOI:** 10.5811/cpcem.2021.6.52893

**Published:** 2022-04-25

**Authors:** Christopher Trumbetta, Michael Galuska

**Affiliations:** Conemaugh Memorial Medical Center, Department of Emergency Medicine, Johnstown, Pennsylvania

**Keywords:** conducted energy weapon, Brugada syndrome, electrocardiogram

## Abstract

**Introduction:**

A 38-year-old with suicidal ideation and alcohol intoxication received conducted energy from a conducted energy weapon (CEW) and subsequently was found to have a transient electrocardiogram (ECG) abnormality consistent with Brugada waveform that resolved over a period of three hours.

**Case Report:**

A 38-year-old male with no pertinent medical history presented with suicidal ideation and alcohol intoxication after an altercation with the police. The patient received two CEW exposures during an encounter with law enforcement prior to transport to the emergency department. He was asymptomatic, but an ECG was performed as part of the triage process given his reported CEW exposure. His initial ECG showed ST-segment and T-wave changes in the precordial leads similar to those found in Brugada syndrome. After a three-hour period of observation and resolution of the patient’s alcohol intoxication, a repeat ECG was performed that showed resolving Brugada morphology.

**Conclusion:**

Review of the literature surrounding the safety profile associated with CEW exposure shows few if any documented concerning cardiac electrophysiology changes and suggests that routine electrocardiographic studies or monitoring is not required. This case presents an isolated but interesting instance of a transient ECG abnormality associated with a CEW exposure.

## INTRODUCTION

Exposure to a conducted energy weapon (CEW) in the field has been shown to result in little risk of serious medical injury to patients in multiple studies conducted over the past 15 years.^1^ Two recent meta-analyses including 37 appropriate articles and studies revealed zero cases of significant electrocardiographic changes after exposure to a CEW.^1^,^2^ These studies and the subsequent reviews have led to recommendations that no routine electrocardiogram (ECG) or cardiac enzyme evaluation are indicated in asymptomatic patients after CEW exposure. With this prior evidence in mind, we present a case of an asymptomatic patient status post CEW exposure with an ECG revealing significant electrocardiographic changes similar in morphology to Brugada syndrome.

## CASE REPORT

A 38-year-old male with a past medical history of mild, intermittent asthma presented with suicidal ideation and alcohol intoxication. Reports from the local police department transporting the patient to the emergency department (ED) indicated he was uncooperative in the field and required two CEW exposures for officers to safely transport him. The patient did admit to telling police to “end his life” by shooting him but also admitted to alcohol consumption earlier in the evening and a domestic dispute causing him to be emotional. He did regret those statements and denied any suicidal or homicidal ideation at time of initial evaluation. The patient denied any symptoms of chest pain and noted no family history of sudden cardiac death.

**Image 1:**
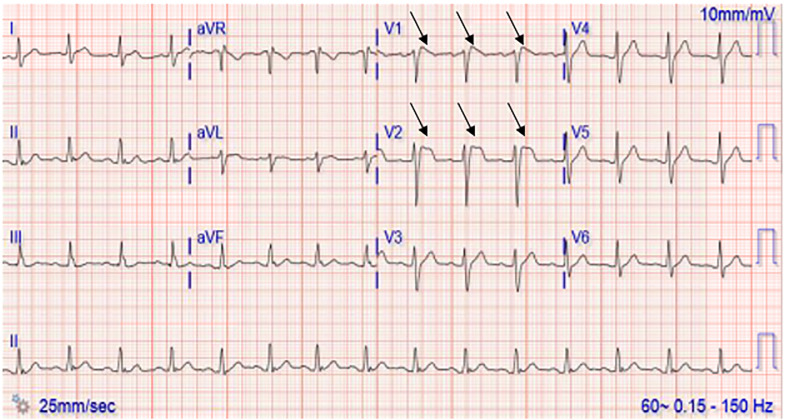
Patient’s initial electrocardiogram showing ST-segment and T-wave changes in leads V1 and V2 (arrows) consistent with a Brugada type II pattern without a prior for comparison.

Initial evaluation included a negative physical examination. An ECG performed as part of the triage process, given the reported CEW exposure, revealed Brugada type II pattern ST-segment and T wave changes in precordial leads (Image 1). This initial ECG was performed approximately one hour post CEW exposure. Laboratory studies included a negative complete blood count, complete metabolic panel, and troponin. Laboratory studies were significant for an ethanol level of 143 milligrams per deciliter (mg/dL) (reference: less than 10 mg/dL).

**Image 2:**
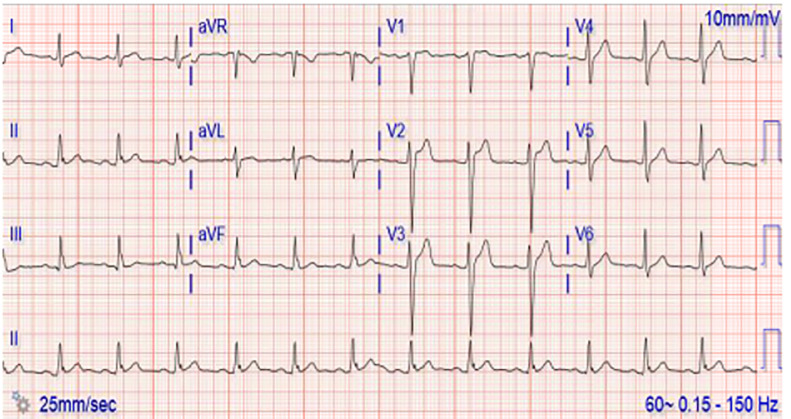
Patient’s repeat electrocardiogram after a three-hour observation period showing persistent J point elevation but resolving ST-segment “saddleback” elevation in leads V1 and V2.

The patient was observed in the ED under suicide precautions until he was no longer intoxicated. A repeat ECG after the three-hour observation period showed improvement and resolving Brugada-like changes noted previously (Image 2). He continued to be physically asymptomatic and denied suicidal or homicidal ideation and was able to demonstrate clear and coherent thought. Psychiatry was consulted, and after evaluation determined that the patient was no longer intoxicated and was competent to make his own medical decisions. The psychiatry consult also determined that he was not a significant danger to himself or anyone else and recommended further evaluation with psychiatry on an outpatient basis. At that time, he was discharged to home in stable condition with psychiatry and family practice follow-up.

## DISCUSSION

Evaluation of patients after encounters with law enforcement is common in the ED. On certain occasions, patients are exposed to CEWs to be safely subdued before transport to the ED. A CEW causes pain and temporary neuromuscular incapacitation by delivering a closed circuit of energy to the patient by direct contact of two electrode darts.^4^ This energy is delivered for a predetermined period during which the energy causes involuntary skeletal muscle contraction and temporary incapacitation, allowing the patient to be safely restrained.^5^

**CPC-EM Capsule**

What do we already know about this clinical entity?

*The use of conducted electrical weapons (CEW) to subdue persons in the field has largely been found to be safe, specifically regarding cardiovascular physiology*.

What makes this presentation of disease reportable?

*Recent meta-analysis reviews have found very little evidence of electrocardiographic changes documented after CEW exposure*.

What is the major learning point?

*This is an isolated but potentially significant case of a transient Brugada type electrocardiographic pattern in a patient after CEW exposure*.

How might this improve emergency medicine practice?

*This case may lead the emergency physician to at least consider cardiac electrophysiology pathologies after CEW exposure in the appropriate clinical setting*.

Early studies of the safety of CEW exposure included investigation of whether the energy could potentiate cardiac dysrhythmias.^2^ There were isolated case reports of cardiac dysrhythmias following CEW exposure, including one from a prehospital report in 2005 in which a young, healthy patient was found to be in cardiopulmonary arrest with a ventricular fibrillation rhythm shortly after exposure.^6^ However, numerous studies investigating the safety of CEW exposure have demonstrated little to no cardiovascular risk and few, if any, documented cardiac dysrhythmias. This finding was confirmed in a recent meta-analysis by Vilke et al^1^ that reviewed the results of 37 studies conducted from 1998–2018 on the safety of CEW exposure and found zero incidence of immediate or delayed cardiac ischemia or dysrhythmias.^1^ Based on their meta-analysis, the authors advised against routine electrocardiographic study on an asymptomatic patient with recent CEW exposure.^1^

Here we present a case in which transient ECG changes suggestive of an abnormal QRS conduction consistent with Brugada pattern were found shortly after CEW exposure. Brugada syndrome is a genetic condition most commonly associated with sodium channelopathies that is linked to polymorphic ventricular tachycardias and sudden cardiac death.^7^ This is not to be confused with a Brugada pattern, which is described as ECG findings not associated with any cardiac symptoms.^7^ The ECG findings classically described with a Brugada pattern are ST-T segment changes in V1 and V2 generally broken down into two types, I and II.^8^ Type I demonstrates a “coved,” elevated but downward sloping ST segment that transitions into an inverted T wave, while type II demonstrates a “saddleback” elevated ST segment with either an upright or inverted T wave.^9^ The ECG changes in this case most closely represent a type II Brugada pattern, with clearly identifiable “saddleback” ST-segment elevation in lead V2 of Image 1. While lead V1 of image 1 does appear to have a more “coved” appearance to the ST-segment elevation, there is no clear T-wave inversion, a distinctive feature of a type I Brugada pattern.

The patient who presented with Brugada-type waveform documented in this case did have an initial ethanol level of 143 mg/dL but was calm, cooperative, oriented to person, place, month and date, and had no clinical signs of acute intoxication and a negative urine drug analysis. He had no family history of sudden cardiac death and his presentation was not associated with any clinical signs or symptoms of cardiac ischemia or elevation in serum cardiac biomarkers. In addition, the ECG changes spontaneously improved and began resolving after a period of observation, and no ventricular dysrhythmias were noted while the patient was observed on the cardiac monitor while in the ED. It is worth noting that there are several other well documented causes of transient Brugada patterns including ethanol ingestion, cocaine ingestion, cardiac ischemia, and fever. In addition, the current literature on CEW exposure supports the notion that cardiac dysrhythmias are rare. While our patient did develop a transient Brugada pattern, we never identified a cardiac dysrhythmia. Nonetheless, the temporal relationship with recent CEW exposure and transient Brugada pattern is interesting and worth adding to the evolving picture of the safety profile associated with these weapons.

## CONCLUSION

Evaluation of patients in the ED setting after exposure to a conducted electrical weapon is common. Studies over the past 20 years on the potential harmful effects of CEW exposure suggest that no cardiovascular pathology is imposed on the patient and that routine electrocardiographic studies or monitoring are not required. The case presented here is an isolated but interesting instance of a transient ECG abnormality associated with CEW exposure.
